# Mutant p53 – Heat Shock Response Oncogenic Cooperation: A New Mechanism of Cancer Cell Survival

**DOI:** 10.3389/fendo.2015.00053

**Published:** 2015-04-22

**Authors:** Evguenia M. Alexandrova, Natalia D. Marchenko

**Affiliations:** ^1^Department of Pathology, Stony Brook University, Stony Brook, NY, USA

**Keywords:** mutant p53, GOF, heat shock response, HSF1, Her2, Neu, EGFR

## Abstract

The main tumor suppressor function of p53 as a “guardian of the genome” is to respond to cellular stress by transcriptional activation of apoptosis, growth arrest, or senescence in damaged cells. Not surprisingly, mutations in the p53 gene are the most frequent genetic alteration in human cancers. Importantly, mutant p53 (mutp53) proteins not only lose their wild-type tumor suppressor activity but also can actively promote tumor development. Two main mechanisms accounting for mutp53 proto-oncogenic activity are inhibition of the wild-type p53 in a dominant-negative fashion and gain of additional oncogenic activities known as gain-of-function (GOF). Here, we discuss a novel mechanism of mutp53 GOF, which relies on its oncogenic cooperation with the heat shock machinery. This coordinated adaptive mechanism renders cancer cells more resistant to proteotoxic stress and provides both, a strong survival advantage to cancer cells and a promising means for therapeutic intervention.

## Oncogenic Functions of Mutant p53

Mutations in the p53 tumor suppressor gene are the most frequent tumor-associated genetic alterations throughout the entire spectrum of human cancers (1). In contrast to other tumor suppressors that are commonly inactivated by frameshift and nonsense mutations resulting in loss-of-function, the majority of p53 alterations are missense mutations clustered in six “hot-spots” of the DNA-binding domain of p53 (2). Numerous mouse models, *in vitro* and clinical studies have demonstrated that in addition to simple loss of the tumor suppressor function of p53, many mutant p53 (mutp53) proteins gain neomorphic oncogenic activities, termed as gain-of-function (GOF) (2, 3). These GOF activities contribute to malignant transformation by enhancing cells proliferation, invasion, metastatic ability, and chemoresistance (2, 3). The concept of mutp53 GOF is strongly supported by human clinical studies on Li–Fraumeni syndrome (LFS) patients carrying germline p53 mutations and by GOF mouse models (4–7). Thus, several studies have found that the median age of cancer onset in LFS patients with mutp53 missense mutations is 9–20 years earlier than in LFS patients with loss of p53 expression (8, 9). Moreover, clinical evaluation of 1,794 breast cancer patients revealed that *somatic* p53 mutations are also associated with a shorter overall survival, independently of stage, grade, and hormone receptors status (10), similarly to other cancer types harboring mutp53 (1). This is fully confirmed by mouse mutp53 knock-in models, manifesting GOF by increased metastases, broader tumor spectrum, more invasive tumor fronts, more malignant histology, and higher tumor bulk compared to p53-null tumors (4–7).

A broad spectrum of GOF activities has been described for mutp53 [reviewed in Ref. (2, 3)]. Among the most prominent ones is the ability of mutp53 to promote cells proliferation, invasion, and motility by stimulating signal transduction pathways downstream of growth factor receptors, such as TGFβ receptor (11), EGFR (4, 12, 13), MET (14), PDGFRβ (15), as well as ErbB2/Her2 (4, 13) discussed below. Also, a number of *in vitro* and *in vivo* studies have described a critical role of mutp53 in tumor initiation via enhanced generation and expansion of cell populations with stem cell/cancer stem cell properties (4, 5, 16, 17). In addition, recent reports indicate that mutp53 promotes the inflammatory response and inflammation-associated cancers by stimulating NF-κB activation (18, 19). Finally, a novel intriguing mutp53 GOF activity has been described in tumor-associated fibroblasts (20, 21), suggesting that mutp53 can play oncogenic roles not only in cancer cells but also in the tumor stromal cells.

Mechanistically, although the majority of mutp53 missense mutations map to its DNA-binding domain, mutp53 GOF activities are still largely attributed to transcriptional regulation of specific target genes, which differ from typical wild-type p53 targets [reviewed in Ref. (2)]. While mutp53-specific DNA consensus sites have not been identified, it appears that mutp53 is a potent modulator of other transcription factors and co-factors, via regulation of their DNA binding and transcriptional properties, such as p63 (11, 12, 22, 23), p73 (22), SP1 (24), SREBP (25), and others [reviewed in Ref. (2, 3)] including the master heat shock regulator HSF1 (13), discussed below.

A critical feature of most mutp53-harboring tumors is significantly increased mutp53 protein stability, manifested by massive mutp53 accumulation in tumors, but not in normal tissues (6, 26). We and others showed that cancer-specific accumulation of mutp53 is crucial for many aspects of tumorigenesis and is the key determinant of mutp53 GOF *in vitro* and *in vivo* (7, 11, 27, 28). Thus, acute downregulation of stabilized mutp53 by RNA interference (RNAi) strongly inhibits malignant phenotypes (11, 27, 28). For example, we found that stable and Tet-inducible knockdown of endogenous mutp53 in breast (MDA231) and colon (SW480) cancer cells by p53 RNAi dramatically inhibits growth of these human cancer cells *in vitro* and in xenografts and their invasive properties (28). These data are consistent with other reports showing that mutp53 downregulation by RNAi suppresses invasion (11, 28), restores normal mammary architecture in 3D culture of breast cancer cell lines (25), and inhibits metastasis *in vivo* (11, 15). Thus, cancer cells appear to be addicted to high levels of mutp53 for their survival and oncogenic properties.

Addiction of cancer cells to stabilized mutp53 underscores the translational significance of mutp53 as a promising therapeutic target. However, targeting mutp53 by conventional modalities is a very challenging task, since mutp53 is neither an enzyme nor a cell surface protein. Therefore, one promising alternative for abolishing mutp53 GOF in mutp53-harboring cancers could be its depletion/destabilization. We recently proposed that this could be achieved by exploiting mutp53 interdependence with the heat shock response machinery (13, 28, 29). Thus, we showed that mutp53 has a novel GOF activity as an essential regulator of protein homeostasis in cancer. Specifically, it augments the pro-survival heat shock response machinery via activating the master transcriptional regulator heat shock factor 1 (HSF1), which in a positive feed-forward loop further stabilizes mutp53 itself, along with other tumor-promoting clients (13). This novel oncogenic GOF activity of mutp53 may represent a unique adaptive mechanism for superior survival of mutp53-harboring cancer cells in the hostile tumor environment.

## Mutant p53 and the Heat Shock Response

Inherent to malignant transformation is the constant proteotoxic stress due to aneuploidy, accumulation of reactive oxygen species (ROS), hypoxia, acidosis, and accumulation of mutated, conformationally aberrant proteins (30–32). To overcome these potentially deadly conditions for their survival, cancer cells heavily depend on molecular chaperones, heat shock proteins (HSPs), whose induction in cancer constitutes the powerful adaptive pro-survival mechanism known as the heat shock response (32). Under proteotoxic stress, induction of HSPs restores protein homeostasis by repairing and proper folding of damaged and mutated proteins with aberrant conformation. HSPs induction in cancer cells is triggered by the transcription factor HSF1 that binds to unique DNA sequence motifs known as heat shock elements (HSEs) in the promoters of HSPs, inducing their transcription (33). In unstressed cells, HSF1 is sequestered by HSP90 predominantly in the cytoplasm (30). However, proteotoxic stress induces HSF1 phosphorylation, liberation from the HSP90 inhibitory complex, trimerization, and translocation to the nucleus to activate HSPs expression (34). It has been shown that phosphorylation of HSF1 at serine 326 (pSer326) is pivotal to render HSF1 transcriptionally competent (30, 34). Also, HSF1 stabilization and activation may be induced by genetic changes such as loss of the neurofibromatosis type 1 (NF1) tumor suppressor gene (35).

The essential role of HSF1 in malignant transformation and progression is well documented in literature. Specifically, HSF1 induces a diverse array of HSP-mediated pro-survival mechanisms, including stabilization of oncogenic clients, altered glucose metabolism and signal transduction, and upregulation of protein translation (4, 32, 35, 36). Interestingly, a recent study by the Linquist group has shown that HSF1 has both distinct and overlapping activities in the maintenance of normal protein homeostasis vs. tumorigenesis (36). In cancer HSF1 orchestrates, a wide range of fundamental cellular processes that are not related to heat shock response but are critical for malignant transformation and maintenance, including cell-cycle control, ribosomal biogenesis, protein translation, and inhibition of apoptosis (36). Importantly, cancer-specific HSF1-bound genes (“HSF1 cancer signature”) were found enriched in the biopsies of human breast, colon, and lung tumors and strongly correlated with poor patient outcomes underscoring the critical role of HSF1 in tumorigenesis (36). In agreement, another clinical study found HSF1 upregulation in 80% of breast cancers, which was also associated with high histologic grade and increased mortality (37). Finally, the pivotal role of HSF1 in tumorigenesis is demonstrated in various animal cancer models. For example, genetic deficiency of HSF1 dramatically reduces mammary tumor formation in the Her2/Neu mouse model (38), tumorigenesis in the DMBA-induced skin carcinogenesis and in the mutp53 mouse models (31).

The connection between mutp53 and heat shock response has been known for nearly two decades (39). Thus, mutp53 and HSP90 (one of the most explored HSF1 transcription targets) were shown to physically interact, which was linked to cancer-specific mutp53 stabilization (28, 29, 39, 40). We and others subsequently demonstrated that pharmacological or RNAi-mediated inhibition of HSP90 leads to ubiquitination and proteasomal degradation of mutp53, mediated by the E3 ubiquitin ligases MDM2 and CHIP (28, 29, 40, 41). Moreover, it is likely that other E3 ligases might be involved as well. Thus, a recent report suggests that arsenic trioxide, a drug used to treat acute promyelocytic leukemia, cooperates with HSP90 inhibitors and promotes mutp53 degradation in tumor cells by the Pirh2 ubiquitin ligase (42). It would be interesting to see whether Pirh2 is inhibited by HSPs during malignant transformation similarly to MDM2 and CHIP (29, 40), leading to mutp53 aberrant stabilization, and fueling of its oncogenic properties.

An important evidence for mutp53-HSP90 oncogenic cooperation comes from the studies showing that HSP90 inhibition shows preferential cytotoxicity in mutp53 – rather than in wild-type p53 or p53null – cancer cells and destabilizes mutp53 (28, 29). *In vivo* studies also provide compelling evidence for mutp53-heat shock response oncogenic cooperation. Thus, HSF1 genetic deficiency prolonged median overall survival of mutp53 mice (R172H) in a dose-dependent manner, from 427 to 470 to >622 days in HSF1^+/+^ vs. HSF1^+/−^ vs. HSF1^−/−^ animals, respectively (31). This strongly suggests that either (i) HSF1/HSPs directly maintain mutp53 levels/activity or (ii) the oncogenicity of mutp53 critically depends on HSF1 and/or HSF1-mediated transcriptional program, or both. Although mutp53 levels were not examined in this study (31), it is tempting to speculate that they were reduced in HSF1-deficient tumors as a result of insufficient transcriptional upregulation of HSP90, leading to restrained malignant transformation. In support of this idea, we previously showed that shRNA-mediated knockdown of HSF1 in mutp53 cancer cells induces rapid destabilization of mutp53 and reduces its half-life, along with reduction of HSP90 levels (29). Further studies are needed to confirm this idea *in vivo* and to test the other possible mechanisms of mutp53-HSF1 oncogenic cooperation. Besides that, it would be interesting to examine whether other HSF1 transcriptional chaperone targets besides HSP90, e.g., HSP70 and HSP27, also can stabilize mutp53 in cancer cells and via what mechanisms. Interestingly, a novel mechanism of mutp53 degradation via chaperone-mediated autophagy was recently reported. It has been shown that heat shock cognate protein 70 (HSC70), a constitutive cytosolic protein (*not* regulated by HSF1), can target mutp53 to lysosomal degradation, but only in non-proliferating tumor cells under the condition of proteasomal and macroautophagy inhibition (43). However, the importance of this mechanism in proliferating cancer cells remains to be elucidated.

## Mutant p53 Induces HSF1 Transcriptional Activity via Her2 and EGFR

Although mechanisms regulating the heat shock response in tumor cells are not fully understood, it is well established that heat shock response strongly depends on the transcriptional activity of HSF1. Stress-activated transcriptionally competent form of HSF1 is a homo-trimer, which can be post-translationally modified resulting in HSF1 nuclear translocation and binding to HSP promoters (27, 30). In response to proteotoxic stress, HSF1 becomes phosphorylated at serine 326, which is essential for HSF1 transcriptional activity (27, 31).

A number of studies suggest that the Her2/EGFR2/Neu signaling pathway is an important activator of HSF1, at least in breast cancer. Thus, Her2 overexpression in MCF7 cells leads to increased HSF1 levels and its trimerization (44). Also, HSF1 is necessary and sufficient for Her2-induced transformation of normal breast epithelial cells MCF10A *in vitro* (45). Finally, HSF1 genetic knockout significantly reduces mammary tumorigenesis in the ErbB2 transgenic mouse model (38). Clinically, the presence of high levels of nuclear HSF1 in Her2-positive mammary tumors correlates with poor patient prognosis (37). Moreover, recent studies demonstrate existence of a linear signaling pathway between Her2 and HSF1 in Her2-positive breast cancer, both *in vitro* (13, 46, 47) and *in vivo* (4, 47). Thus, Schulz et al. showed that Her2 overexpression constitutively activates HSF1, resulting in stabilization of HSP90 clients, such as MIF, AKT, mutp53, and HSF1 itself (47). Moreover, pharmacological inhibition of Her2 strongly suppresses HSF1 activation *in vitro* and in the Her2 mouse transgenic model, which correlates with reduced mammary tumor progression (13, 47). Mechanistically, Her2 signals via the phosphoinositide-3 kinase (PI3K)–AKT axis to induce pSer326 phosphorylation of HSF1 and its transcriptional activity (47).

Intriguingly, the Lindquist group found that activation of HSF1 can be triggered not only by environmental and proteotoxic insults but also by genetic alterations. Thus, loss of the tumor suppressor gene neurofibromatosis type 1 (NF1) increases HSF1 levels and induces HSF1 phosphorylation at Ser326, which depends on dysregulated RAS/MAPK signaling, suggesting a key role of RAS/MAPK pathway in the transcriptional activation of HSF1 (35). Consistently, Stanhill et al. showed that RAS activation induces HSF1-mediated upregulation of HSP70 (48). These findings are strongly supported by clinical data. Thus, elevated expression of HSF1 and pSer326-HSF1 was found in surgical specimens of malignant peripheral nerve sheath tumors driven by loss of NF1 (35). Given the importance of HSF1 in cancer cell physiology (36), it will be interesting to see whether loss of other tumor suppressor genes, especially those leading to MAPK/PI3K pathway activation, could trigger transcriptional activity of HSF1 in different tumor types.

How exactly does mutp53 cooperate with HSF1 in cancer? On one hand, as mentioned above, HSF1 genetic ablation profoundly alleviates tumorigenesis driven by mutp53 *in vivo* (31), which could be due to insufficient levels of HSP90 to support mutp53 accumulation in tumors (29, 39, 40). On the other hand, our recent findings demonstrate that mutp53 is also an important determinant of HSF1 activity. Thus, overexpression of various mutp53 alleles in cancer cell lines leads to upregulation of HSF1, with concurrent increase in heat shock response and prototypical HSP clients (13). Furthermore, we found that this mutp53-HSF1 positive feed-forward loop depends on growth factor receptor signaling, specifically EGFR and Her2 pathways, for HSF1 transcriptional activity (4, 13).

Since mutp53 is known to exert its GOF activities via other growth factor receptor signaling pathways besides Her2 and EGFR (see above), these pathways could also be involved in mutp53-HSF1 oncogenic cooperation. Preliminary indirect evidence of this possibility comes from the finding by the Linquist lab that HSF1 enables cellular transformation of mouse embryo fibroblasts via PDGFRβ signaling (31), which was shown to be positively regulated by mutp53 as well (15). However, it remains to be determined whether other growth factor receptor signaling pathways – besides EGFR and Her2 – can modulate HSF1 activity in a mutp53-dependent manner.

Recently, we found another, more direct mode of the mutp53-HSF1 circuit regulation in cancer cells. Thus, upon proteotoxic stress and nuclear translocation, phospho-activated pSer326-HSF1 interacts (directly or indirectly) with mutp53. Moreover, mutp53 facilitates HSF1 recruitment to specific DNA sites (HSEs) in target gene promoters and augments a broad pro-survival HSF1-induced transcriptional program, including expression of HSPs (13).

Finally, as mentioned above, HSF1 transcriptional activity can be stimulated by genetic alterations in cancer (35). Thus, the well-known ability of mutp53 to induce genomic instability (49) could in theory be another mechanism contributing to HSF1 activation in cancer, which remains to be tested.

**Figure 1:**
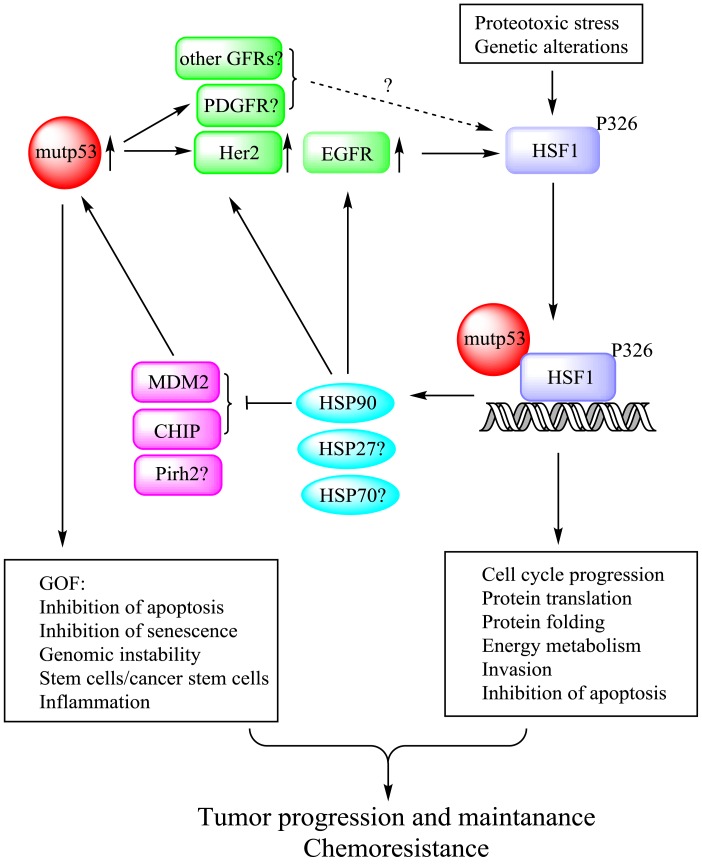
**Mutp53 potentiates HSF1 activation in an EGFR/Her2-dependent manner**. Mutp53, by enhancing EGFR and Her2 signaling, potentiates HSF1 activity via a feed-forward loop and thereby up-regulates HSP90 clients including Her2, EGFR, and mutp53 itself. Thus, mutp53-HSF1 oncogenic liaison endows cancer cells with superior survival and chemoresistance, hence supporting cancer progression and maintenance.

Based on the described findings, we propose a novel mechanism of mutp53 GOF, via regulation of the heat shock response, as depicted in Figure 1. We propose that mutp53 through enhanced recycling (12) and/or stability of EGFR and Her2/ErbB2 – augments MAPK and PI3K signaling, leading to phospho-activation of HSF1. Concurrently, mutp53 directly interacts with activated HSF1 and facilitates its binding to specific promoter elements and stimulates transcription of HSPs. In turn, HSPs further stabilize their oncogenic clients, including EGFR, Her2, and mutp53 itself (via suppression of ubiquitin ligases MDM2, CHIP, etc.), reinforcing tumorigenesis (Figure 1). Importantly, this mechanism has been confirmed by *in vivo* studies. First, clinical data show a strong enrichment (83%) of the Her2-positive breast cancer in LFS women with p53 germline mutations, compared to 20–25% Her2 positivity in patients with sporadic breast cancer (50). This suggests that the presence of mutp53 germline mutations strongly predisposes Li–Fraumeni women specifically to the initiation of Her2-driven breast cancer. Second, we found a strong correlation between mutp53 and nuclear pSer326-HSF1 levels only in strongly Her2-positive (3+), but not in Her2-negative/ER/PR-positive human breast cancer specimens (13). Finally, we demonstrated that the Her2/ErbB2 signaling is much more amplified in mutp53 (R172H) compared to p53null tumor cells in the MMTV-ErbB2 mouse breast cancer model, leading to a more aggressive disease (4).

Many physiological consequences can result from the described mutp53-HSF1 liaison in cancer cells. First, the resulting increased expression of HSPs endows cancer cells with a superior resistance to proteotoxic stress caused by harsh tumor environment (13). Second, it leads to enhanced stabilization of numerous tumor-promoting HSP oncogenic clients, including Her2, EGFR, and mutp53 themselves, which cell-autonomously further amplify this feed-forward circuit and oncogenesis. Third, HSPs have been shown to inhibit oncogene-induced senescence pathways in cancer cells (51). Thus, by enhancing HSP expression at early stages of tumorigenesis, mutp53 may facilitate disabling of oncogene-induced senescence and therefore empower tumor progression. Fourth, we showed that mutp53-HSF1-mediated amplification of Her2 pathway can promote expansion of mammary stem cells and induce cancer cell proliferation *in vivo* (4), which can enhance tumor initiation and progression. Finally, by augmenting the broad HSF1-dependent transcriptional program, mutp53 may promote global cancer-related changes, including cell-cycle progression, altered signaling pathways, metabolism, adhesion, protein translation, etc. (36).

In sum, it is evident that the functional oncogenic interaction between mutp53 and HSF1 can initiate a wide range of tumorigenic processes in the complex landscape of mutp53-harboring cancers.

## Summary

While mutations in the p53 gene are prevailing in many types of cancer, specific therapeutic modalities tailored to mutp53-harboring cancers have not been developed in clinic. Three potential mutp53-targeted therapeutic strategies have been recently proposed: (i) restoration of wild-type p53 activity in mutp53 proteins (3), (ii) inhibition of mutp53-regulated downstream targets and pathways, e.g., proteins involved in integrin recycling (12), the mevalonate pathway (25), PDGFRβ signaling (15), etc. [reviewed in Ref. (3)], and (iii) mutp53 degradation (28, 29, 42). Overall, understanding the mechanisms underlying mutp53 oncogenic activity will no doubt have a profound translational impact. However, more in-depth studies are needed to establish whether these approaches will be clinically feasible and whether pharmacological targeting of relevant pathways will achieve preferential response in the patients with mutp53-harboring tumors.

The herein described novel oncogenic GOF role of mutp53 in the regulation of heat shock response – via enhanced receptor tyrosine kinase (Her2, EGFR) signaling and augmented HSF1 transcriptional activity – opens up novel therapeutic opportunities. We anticipate that the mutp53-HSF1 liaison, due to potentiating Her2 and/or EGFR pathways, sensitizes cancer cells to ErbB2 and EGFR targeted therapies. Thus, inhibition of Her2, EGFR, or their downstream effectors can intercept the sensitive mutp53-HSF1-Her2/EGFR circuitry and therefore be a potent approach for the treatment of Her2 (or EGFR) and mutp53 double-positive cancers. Convergence of the mutp53-HSF1 liaison on the Her2/EGFR pathways provides a strong rationale to test the targeted therapies that are currently on the market (e.g., Her2-targeted trastuzamab, pertuzumab, T-DM1, and lapatinib) specifically in mutp53-harboring cancers. The most promising cancer types expected to specifically respond to these therapies are Her2/mutp53 double-positive breast cancer [constituting 72% of all sporadic Her2-positive breast cancers (52)], pancreatic and non-small-cell lung cancer [both of which have high prevalence of mutp53 (1) and are commonly treated with EGFR inhibitor Erlotinib], and possibly esophageal cancer [43% mutp53-positive (1), 23% Her2-positive]. Although it remains to be determined whether the Her2-positive subtype of esophageal cancers is enriched in mutp53, similarly to breast cancer. Importantly, the data described here also predict that the patients with tumor types that commonly overexpress both, Her2/EGFR and mutp53, will need to be stratified according to their mutp53 status for the most efficient treatment outcomes.

## Conflict of Interest Statement

The authors declare that the research was conducted in the absence of any commercial or financial relationships that could be construed as a potential conflict of interest.

## Abbreviations

EGFR, epidermal growth factor receptor; GOF, gain-of-function; HSE, heat shock elements; HSF1, heat shock factor 1; HSP, heat shock protein; LFS, Li–Fraumeni syndrome; mutp53, mutant p53; NF1, neurofibromatosis type 1; PDGFR, platelet-derived growth factor receptor.

## References

[1] Olivier M Hollstein M Hainaut P. TP53 mutations in human cancers: origins, consequences, and clinical use. Cold Spring Harb Perspect Biol (2010) 2(1):a001008. 10.1101/cshperspect.a001008 20182602 PMC2827900

[2] Brosh R Rotter V. When mutants gain new powers: news from the mutant p53 field. Nat Rev Cancer (2009) 9(10):701–13. 10.1038/nrc2693 19693097

[3] Muller PA Vousden KH Mutant p53 in cancer: new functions and therapeutic opportunities. Cancer Cell (2014) 25(3):304–17 10.1016/j.ccr.2014.01.021 24651012 PMC3970583

[4] Yallowitz AR Li D Lobko A Mott D Nemajerova A Marchenko N. Mutant p53 amplifies epidermal growth factor receptor family signaling to promote mammary tumorigenesis. Mol Cancer Res (2015) 13(4):1–12. 10.1158/1541-7786.MCR-14-0360 25573952 PMC4824060

[5] Hanel W Marchenko N Xu S Yu SX Weng W Moll U. Two hot spot mutant p53 mouse models display differential gain of function in tumorigenesis. Cell Death Differ (2013) 20(7):898–909. 10.1038/cdd.2013.17 23538418 PMC3679454

[6] Lang GA Iwakuma T Suh YA Liu G Rao VA Parant JM Gain of function of a p53 hot spot mutation in a mouse model of Li-Fraumeni syndrome. Cell (2004) 119(6):861–72. 10.1016/j.cell.2004.11.006 15607981

[7] Terzian T Suh YA Iwakuma T Post SM Neumann M Lang GA The inherent instability of mutant p53 is alleviated by Mdm2 or p16INK4a loss. Genes Dev (2008) 22(10):1337–44. 10.1101/gad.1662908 18483220 PMC2377188

[8] Zerdoumi Y Aury-Landas J Bonaïti-Pellié C Derambure C Sesboüé R Renaux-Petel M Drastic effect of germline TP53 missense mutations in Li-Fraumeni patients. Hum Mutat (2013) 34(3):453–61. 10.1002/humu.22254 23172776

[9] Bougeard G Sesboüé R Baert-Desurmont S Vasseur S Martin C Tinat J Molecular basis of the Li-Fraumeni syndrome: an update from the French LFS families. J Med Genet (2008) 45(8):535–8 10.1136/jmg.2008.057570 18511570

[10] Olivier M Langerød A Carrieri P Bergh J Klaar S Eyfjord J The clinical value of somatic TP53 gene mutations in 1,794 patients with breast cancer. Clin Cancer Res (2006) 12(4):1157–67. 10.1158/1078-0432.CCR-05-1029 16489069

[11] Adorno M Cordenonsi M Montagner M Dupont S Wong C Hann B A Mutant-p53/Smad complex opposes p63 to empower TGFbeta-induced metastasis. Cell (2009) 137(1):87–98. 10.1016/j.cell.2009.01.039 19345189

[12] Muller PA Caswell PT Doyle B Iwanicki MP Tan EH Karim S Mutant p53 drives invasion by promoting integrin recycling. Cell (2009) 139(7):1327–41. 10.1016/j.cell.2009.11.026 20064378

[13] Li D Yallowitz A Ozog L Marchenko N. A gain-of-function mutant p53-HSF1 feed forward circuit governs adaptation of cancer cells to proteotoxic stress. Cell Death Dis (2014) 5:e1194. 10.1038/cddis.2014.158 24763051 PMC4001312

[14] Muller PA Trinidad AG Timpson P Morton JP Zanivan S van den Berghe PV Mutant p53 enhances MET trafficking and signalling to drive cell scattering and invasion. Oncogene (2013) 32(10):1252–65. 10.1038/onc.2012.148 22580601 PMC3592945

[15] Weissmueller S Manchado E Saborowski M Morris JP IV Wagenblast E Davis CA Mutant p53 drives pancreatic cancer metastasis through cell-autonomous PDGF receptor beta signaling. Cell (2014) 157(2):382–94. 10.1016/j.cell.2014.01.066 24725405 PMC4001090

[16] Lu X Liu DP Xu Y. The gain of function of p53 cancer mutant in promoting mammary tumorigenesis. Oncogene (2012) 32(23):2900–6. 10.1038/onc.2012.299 22824795 PMC3586389

[17] Sarig R Rivlin N Brosh R Bornstein C Kamer I Ezra O Mutant p53 facilitates somatic cell reprogramming and augments the malignant potential of reprogrammed cells. J Exp Med (2010) 207(10):2127–40. 10.1084/jem.20100797 20696700 PMC2947075

[18] Cooks T Pateras IS Tarcic O Solomon H Schetter AJ Wilder S Mutant p53 prolongs NF-kappaB activation and promotes chronic inflammation and inflammation-associated colorectal cancer. Cancer Cell (2013) 23(5):634–46. 10.1016/j.ccr.2013.03.022 23680148 PMC3657134

[19] Di Minin G Bellazzo A Dal Ferro M Chiaruttini G Nuzzo S Bicciato S Mutant p53 reprograms TNF signaling in cancer cells through interaction with the tumor suppressor DAB2IP. Mol Cell (2014) 56(5):617–29. 10.1016/j.molcel.2014.10.013 25454946

[20] Madar S Harel E Goldstein I Stein Y Kogan-Sakin I Kamer I Mutant p53 attenuates the anti-tumorigenic activity of fibroblasts-secreted interferon beta. PLoS One (2013) 8(4):e61353. 10.1371/journal.pone.0061353 23630584 PMC3632588

[21] Addadi Y Moskovits N Granot D Lozano G Carmi Y Apte RN p53 status in stromal fibroblasts modulates tumor growth in an SDF1-dependent manner. Cancer Res (2010) 70(23):9650–8. 10.1158/0008-5472.CAN-10-1146 20952507 PMC2999653

[22] Gaiddon C Lokshin M Ahn J Zhang T Prives C. A subset of tumor-derived mutant forms of p53 down-regulate p63 and p73 through a direct interaction with the p53 core domain. Mol Cell Biol (2001) 21(5):1874–87. 10.1128/MCB.21.5.1874-1887.2001 11238924 PMC86759

[23] Muller PA Trinidad AG Caswell PT Norman JC Vousden KH. Mutant p53 regulates dicer through p63-dependent and -independent mechanisms to promote an invasive phenotype. J Biol Chem (2014) 289(1):122–32. 10.1074/jbc.M113.502138 24220032 PMC3879536

[24] Chicas A Molina P Bargonetti J. Mutant p53 forms a complex with Sp1 on HIV-LTR DNA. Biochem Biophys Res Commun (2000) 279(2):383–90. 10.1006/bbrc.2000.3965 11118296

[25] Freed-Pastor WA Mizuno H Zhao X Langerød A Moon SH Rodriguez-Barrueco R Mutant p53 disrupts mammary tissue architecture via the mevalonate pathway. Cell (2012) 148(1–2):244–58. 10.1016/j.cell.2011.12.017 22265415 PMC3511889

[26] Rotter V. p53, a transformation-related cellular-encoded protein, can be used as a biochemical marker for the detection of primary mouse tumor cells. Proc Natl Acad Sci U S A (1983) 80(9):2613–7. 10.1073/pnas.80.9.2613 6189126 PMC393877

[27] Yan W Liu G Scoumanne A Chen X. Suppression of inhibitor of differentiation 2, a target of mutant p53, is required for gain-of-function mutations. Cancer Res (2008) 68(16):6789–96. 10.1158/0008-5472.CAN-08-0810 18701504 PMC2597213

[28] Li D Marchenko ND Moll UM. SAHA shows preferential cytotoxicity in mutant p53 cancer cells by destabilizing mutant p53 through inhibition of the HDAC6-Hsp90 chaperone axis. Cell Death Differ (2011) 18(12):1904–13. 10.1038/cdd.2011.71 21637290 PMC3170683

[29] Li D Marchenko ND Schulz R Fischer V Velasco-Hernandez T Talos F Functional inactivation of endogenous MDM2 and CHIP by HSP90 causes aberrant stabilization of mutant p53 in human cancer cells. Mol Cancer Res (2011) 9(5):577–88. 10.1158/1541-7786.MCR-10-0534 21478269 PMC3097033

[30] Ciocca DR Arrigo AP Calderwood SK. Heat shock proteins and heat shock factor 1 in carcinogenesis and tumor development: an update. Arch Toxicol (2013) 87(1):19–48. 10.1007/s00204-012-0918-z 22885793 PMC3905791

[31] Dai C Whitesell L Rogers AB Lindquist S Heat shock factor 1 is a powerful multifaceted modifier of carcinogenesis. Cell (2007) 130(6):1005–18 10.1016/j.cell.2007.07.020 17889646 PMC2586609

[32] Whitesell L Lindquist S. Inhibiting the transcription factor HSF1 as an anticancer strategy. Expert Opin Ther Targets (2009) 13(4):469–78. 10.1517/14728220902832697 19335068

[33] Sakurai H Enoki Y. Novel aspects of heat shock factors: DNA recognition, chromatin modulation and gene expression. FEBS J (2010) 277(20):4140–9. 10.1111/j.1742-4658.2010.07829.x 20945530

[34] Sarge KD Murphy SP Morimoto RI. Activation of heat shock gene transcription by heat shock factor 1 involves oligomerization, acquisition of DNA-binding activity, and nuclear localization and can occur in the absence of stress. Mol Cell Biol (1993) 13(3):1392–407. 8441385 10.1128/mcb.13.3.1392-1407.1993 PMC359449

[35] Dai C Santagata S Tang Z Shi J Cao J Kwon H Loss of tumor suppressor NF1 activates HSF1 to promote carcinogenesis. J Clin Invest (2012) 122(10):3742–54. 10.1172/JCI62727 22945628 PMC3461912

[36] Mendillo ML Santagata S Koeva M Bell GW Hu R Tamimi RM HSF1 drives a transcriptional program distinct from heat shock to support highly malignant human cancers. Cell (2012) 150(3):549–62. 10.1016/j.cell.2012.06.031 22863008 PMC3438889

[37] Santagata S Hu R Lin NU Mendillo ML Collins LC Hankinson SE High levels of nuclear heat-shock factor 1 (HSF1) are associated with poor prognosis in breast cancer. Proc Natl Acad Sci U S A (2011) 108(45):18378–83. 10.1073/pnas.1115031108 22042860 PMC3215027

[38] Xi C Hu Y Buckhaults P Moskophidis D Mivechi NF. Heat shock factor Hsf1 cooperates with ErbB2 (Her2/Neu) protein to promote mammary tumorigenesis and metastasis. J Biol Chem (2012) 287(42):35646–57. 10.1074/jbc.M112.377481 22847003 PMC3471706

[39] Blagosklonny MV Toretsky J Bohen S Neckers L. Mutant conformation of p53 translated in vitro or in vivo requires functional HSP90. Proc Natl Acad Sci U S A (1996) 93(16):8379–83. 10.1073/pnas.93.16.8379 8710879 PMC38679

[40] Peng Y Chen L Li C Lu W Chen J. Inhibition of MDM2 by hsp90 contributes to mutant p53 stabilization. J Biol Chem (2001) 276(44):40583–90. 10.1074/jbc.M102817200 11507088

[41] Whitesell L Sutphin PD Pulcini EJ Martinez JD Cook PH. The physical association of multiple molecular chaperone proteins with mutant p53 is altered by geldanamycin, an hsp90-binding agent. Mol Cell Biol (1998) 18(3):1517–24. 9488468 10.1128/mcb.18.3.1517 PMC108866

[42] Yan W Jung YS Zhang Y Chen X. Arsenic trioxide reactivates proteasome-dependent degradation of mutant p53 protein in cancer cells in part via enhanced expression of Pirh2 E3 ligase. PLoS One (2014) 9(8):e103497. 10.1371/journal.pone.0103497 25116336 PMC4130519

[43] Vakifahmetoglu-Norberg H Kim M Xia HG Iwanicki MP Ofengeim D Coloff JL Chaperone-mediated autophagy degrades mutant p53. Genes Dev (2013) 27(15):1718–30. 10.1101/gad.220897.113 23913924 PMC3744729

[44] Zhao YH Zhou M Liu H Ding Y Khong HT Yu D Upregulation of lactate dehydrogenase A by ErbB2 through heat shock factor 1 promotes breast cancer cell glycolysis and growth. Oncogene (2009) 28(42):3689–701. 10.1038/onc.2009.229 19668225

[45] Meng L Gabai VL Sherman MY. Heat-shock transcription factor HSF1 has a critical role in human epidermal growth factor receptor-2-induced cellular transformation and tumorigenesis. Oncogene (2010) 29(37):5204–13. 10.1038/onc.2010.277 20622894 PMC2940982

[46] Khaleque MA Bharti A Sawyer D Gong J Benjamin IJ Stevenson MA Induction of heat shock proteins by heregulin beta1 leads to protection from apoptosis and anchorage-independent growth. Oncogene (2005) 24(43):6564–73. 16007186 10.1038/sj.onc.1208798

[47] Schulz R Streller F Scheel AH Rüschoff J Reinert MC Dobbelstein M HER2/ErbB2 activates HSF1 and thereby controls HSP90 clients including MIF in HER2-overexpressing breast cancer. Cell Death Dis (2014) 5:e980. 10.1038/cddis.2013.508 24384723 PMC4040658

[48] Stanhill A Levin V Hendel A Shachar I Kazanov D Arber N Ha-ras(val12) induces HSP70b transcription via the HSE/HSF1 system, but HSP70b expression is suppressed in Ha-ras(val12)-transformed cells. Oncogene (2006) 25(10):1485–95. 10.1038/sj.onc.1209193 16278678

[49] Song H Hollstein M Xu Y. p53 gain-of-function cancer mutants induce genetic instability by inactivating ATM. Nat Cell Biol (2007) 9(5):573–80. 10.1038/ncb1571 17417627

[50] Masciari S Dillon DA Rath M Robson M Weitzel JN Balmana J Breast cancer phenotype in women with TP53 germline mutations: a Li-Fraumeni syndrome consortium effort. Breast Cancer Res Treat (2012) 133(3):1125–30. 10.1007/s10549-012-1993-9 22392042 PMC3709568

[51] Gabai VL Yaglom JA Waldman T Sherman MY. Heat shock protein Hsp72 controls oncogene-induced senescence pathways in cancer cells. Mol Cell Biol (2009) 29(2):559–69. 10.1128/MCB.01041-08 19001088 PMC2612502

[52] Cancer Genome Atlas N Comprehensive molecular portraits of human breast tumours. Nature (2012) 490(7418):61–70 10.1038/nature11412 23000897 PMC3465532

